# Hydration and primary headaches in children and adolescents: a prospective interventional study

**DOI:** 10.1136/bmjnph-2025-001341

**Published:** 2026-01-19

**Authors:** Patricija Kunstek, Neli Bizjak, Damjan Osredkar, Uroš Godnov, Mary Fewtrell, Evgen Benedik

**Affiliations:** 1 University Children’s Hospital Ljubljana, University Medical Centre Ljubljana, Ljubljana, Slovenia; 2 Biotechnical Faculty, University of Ljubljana, Ljubljana, Slovenia; 3 Medical Faculty, University of Ljubljana, Ljubljana, Slovenia; 4 Faculty of Mathematics, Natural Sciences and Information Technologies, University of Primorska, Koper, Slovenia; 5 UCL Great Ormond Street Institute of Child Health, London, UK

**Keywords:** Dietary patterns, Nutritional treatment, Nutrition assessment, Preventive counselling, Cognitive performance

## Abstract

**Objective:**

Primary headaches are a prevalent health concern among children and adolescents, often affecting their quality of life. Emerging evidence suggests that hydration status may play a role in primary headache occurrence and severity. This study aimed to evaluate the impact of hydration on headache characteristics in children and adolescents with primary headaches.

**Methods:**

A total of 60 children and adolescents diagnosed with primary headaches were enrolled from the outpatient Headache Clinic of the Department of Paediatric Neurology at the University Children’s Hospital, University Medical Centre Ljubljana, Slovenia, between May and September 2023. Participants received personalised hydration recommendations based on established Slovenian dietary guidelines and guidance on appropriate fluid choices. The primary outcome was headache frequency, intensity, and duration, measured at baseline and after the four-month intervention using a headache questionnaire. Fluid intake was assessed using a Food Frequency Questionnaire, while hydration status was evaluated in a sub-group (n=28) using bioelectrical impedance analysis.

**Results:**

Following the four-month intervention, participants demonstrated a significant increase in daily fluid intake from 5–8 glasses/day (one glass=200 mL) to 8–10 glasses/day (p<0.001), which was associated with a reduction in primary headache frequency (1–2 times/week to 3 times/month; p<0.001) and headache intensity (7/10–5/10; p<0.001). However, no significant effect was observed on headache duration (3 hours/headache to 2 hours/headache; p=0.19). Following the intervention, hydration status improved, as evidenced by significant increases in median total body water and extracellular water (p<0.001 and p=0.007, respectively), while the total body water to extracellular water ratio remained unchanged.

**Conclusions:**

This study provides novel evidence supporting adequate fluid intake and hydration as a non-pharmacological intervention for reducing primary headache frequency and intensity in paediatric populations.

**WHAT IS ALREADY KNOWN ON THIS TOPIC**

- Primary headaches are common among children and adolescents and can significantly impact their quality of life.
- Previous studies have suggested a potential link between hydration and headache symptoms, but evidence in paediatric populations has been limited and mostly observational.

**WHAT THIS STUDY ADDS**

- This is the first interventional study to assess the impact of hydration on primary headaches in children and adolescents.
- Personalised hydration guidance significantly increased fluid intake, which was associated with a reduction in headache frequency and intensity.
- Objective hydration assessments using bioelectrical impedance analysis confirmed improvements in hydration status after intervention.

**HOW THIS STUDY MIGHT AFFECT RESEARCH, PRACTICE OR POLICY**

- The findings support hydration as a simple, non-pharmacological strategy for managing primary headaches in children and adolescents.
- Routine assessment and promotion of fluid intake should be considered in clinical practice.
- These results provide a foundation for future research exploring hydration-focused interventions and may inform updates to clinical guidelines and public health policies in paediatric headache management.

## Introduction

 Primary headaches are one of the most common neurological disorders in the paediatric population. They are benign and not a result of disease or other medical conditions. The most common types of primary headaches are migraines and tension-type headaches.^1^ Epidemiological studies estimate that the prevalence of primary headaches in children and adolescents ranges between 40 and 60%, increasing with age^2^ and peaking between ages of 11 and 13.^3^ Primary headaches, from now on headaches, can negatively impact a child’s daily functioning, as pain often leads to school absences, difficulties in concentration and learning, and reduced participation in extracurricular activities.^4^ Among various potential triggers, dehydration has been increasingly recognised as a contributing factor to headaches.^2^ Clinical practice also suggests that adequate fluid intake may play an important role in headache reduction. The importance of proper hydration is emphasised in guidelines for the non-pharmacological management of headaches.^5^

Most studies on hydration and headaches have focused only on adults,^6–11^ leaving a gap in understanding how hydration influences headaches in children and adolescents.

The aim of our study was to investigate whether improving fluid intake and hydration in children and adolescents with headaches could reduce the frequency, duration and intensity of headaches. We assessed their baseline hydration status, implemented a four-month targeted intervention to enhance fluid intake and evaluated its impact on headache characteristics. Our goal was to provide evidence-based insights into hydration as a potential preventive strategy for headaches in this age group.

## Methods

### Design

**Figure 1:**
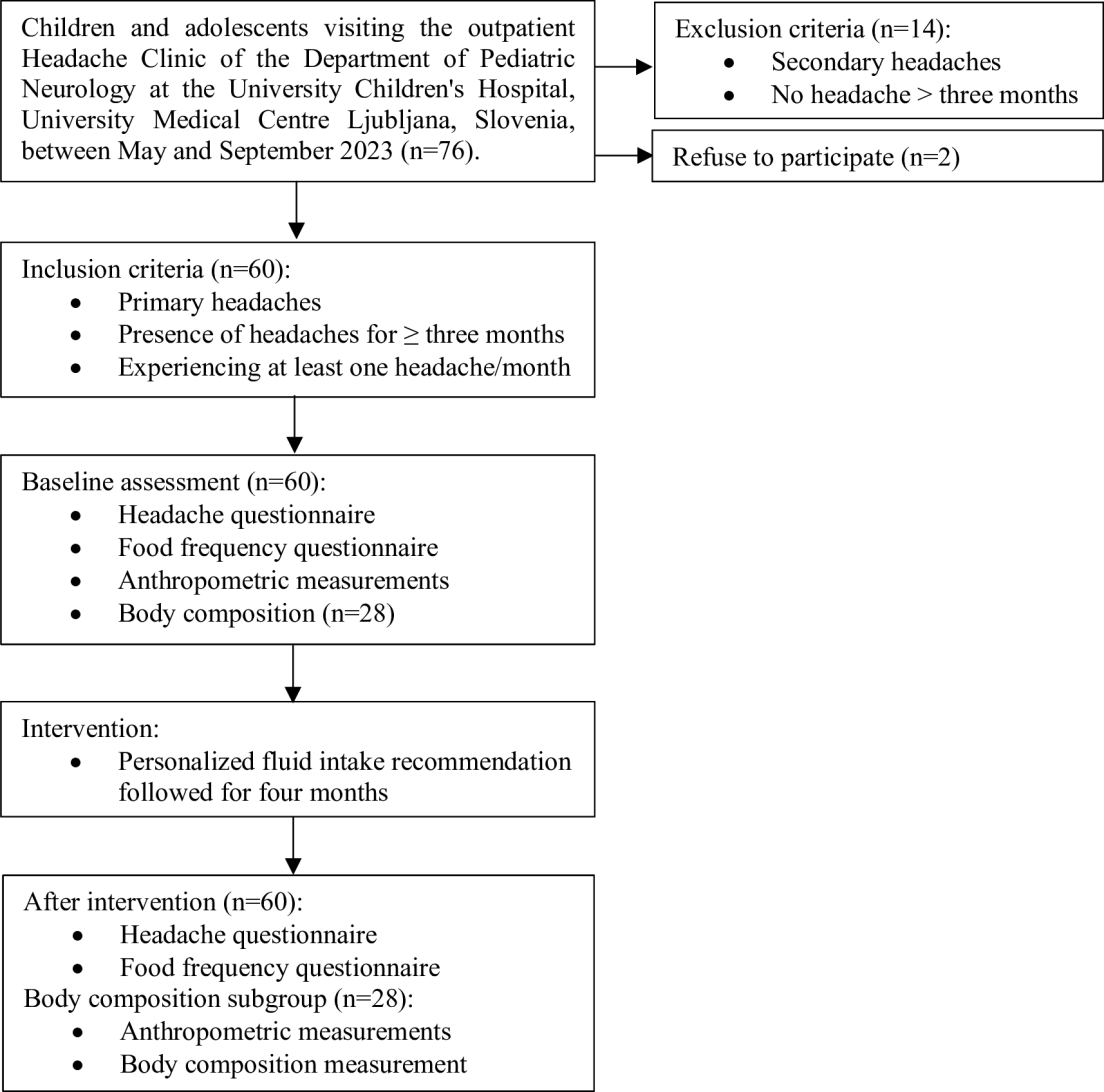
Study flowchart

We conducted a prospective cohort intervention study with a follow-up after four months to evaluate the impact of hydration on headaches in children and adolescents attending the outpatient Headache Clinic of the Department of Paediatric Neurology at the University Children’s Hospital, University Medical Centre Ljubljana, Slovenia. Each participant received personalised daily fluid intake recommendations^12^ from a clinical dietitian, which they followed for four months. Primary outcomes were changes in headache frequency, duration and intensity, assessed using an adapted headache questionnaire.^13^ Secondary outcomes included changes in hydration status, measured using bioelectrical impedance analysis (BIA) in a sub-group. Fluid intake was assessed through a food frequency questionnaire (FFQ).^14^ Additionally, anthropometric measurements (body weight and body height) were recorded at baseline and follow-up. The study flowchart is shown in figure 1. Ethical approval for the study was granted by the National Medical Ethics Committee of the Republic of Slovenia (KME:0120-423/2023-2711-6) and it was registered in Clinical Trials (NCT06816849).

### Participants

The study included children and adolescents, aged 5–19 years, with primary headaches, who were either attending their first or follow-up appointment. Participation was voluntary. Written consent was obtained from guardians and participants aged 15 or older. Only individuals meeting inclusion criteria (primary headaches, presence of headaches for at least three months or more and experiencing at least one headache/month) were included. Exclusion criteria comprised secondary headaches and an absence of headaches for three months or longer. All patients first have the examination done by a paediatric neurologist as part of routine headache management. During the examination, all participants received general lifestyle advice, such as adequate hydration, consuming regular meals, obtaining sufficient sleep, engaging in physical activity and reducing screen time. They were also advised to take an analgesic (most commonly paracetamol or a non-steroidal anti-inflammatory drug, excluding acetylsalicylic acid) in case of severe headache, preferably at its onset. However, they were cautioned not to exceed two or ten analgesics/week.

### Baseline assessment

After the examination by paediatric neurologist, participants who fulfil the inclusion criteria completed a headache questionnaire adapted from Bonfert *et al*,^13^ which assessed headache onset, type, frequency, triggers, duration, headache intensity using a Visual Analogue Scale (VAS) from 1 to 10, associated symptoms (nausea, tingling, phonophobia, photophobia, vomiting, dizziness), family history and lifestyle factors such as fluid intake, meal skipping, physical activity, sleep and electronic device usage in the last month. However, this paper focuses solely on the results related to hydration in relation to headaches.

Fluid intake was assessed using a FFQ, which was previously validated in children within the SI.Menu study.^14^ The FFQ included questions on meal frequency during weekdays and weekends, as well as the consumption of various food and beverages categories. Fluid intake was quantified using the question: “On average, how many glasses (one glass=200 mL) of fluid do you consume/day?” Additionally, the frequency of consumption of various beverage types, including water, mineral water, fruit juice, carbonated and non-carbonated drinks, fruit and herbal teas, coffee, energy drinks, artificially sweetened beverages and alcohol, was assessed. The frequency of each beverage consumption was categorised as follows: never, 1–3 times/month or less, once a week, 2–3 times/week, 4–6 times/week, 1–2 times/day or more. The reported consumption frequencies were converted into numerical frequencies based on predefined calculations (never was converted to 0, 1–3 times/month or less was calculated to 2.0/30=0.07, once a week to 1.0/7=0.14, 2–3 times/week to 2.5/7=0.36, 4–6 times/week to 5.0/7=0.71, 1–2 times/day or more to 1), which allowed for a more precise quantification of beverage intake. The total consumption frequencies of unsweetened beverages and sugar-sweetened beverages (SSBs) were calculated by summing the respective frequencies of individual beverages within each category.

Anthropometric measurements, including body height and body weight, were taken using a digital scale (Seca 220, Germany) with an accuracy of one decimal, and body mass index (BMI) was calculated. The values were later converted into z-scores using combined WHO growth charts and the growth charts of the United Kingdom of Great Britain and Northern Ireland, available at https://www.rcpch.ac.uk.

Body composition was assessed using the BIA 101 analyser (Akern, Italy). BIA measurements were obtained only from participants who consented to body composition analysis at both baseline and at the four month follow-up. The follow-up BIA required an in-person clinic visit, and several families did not return due to travel distance or challenges in arranging an additional visit. As a result, paired BIA data were available for 28 participants.

Key hydration-related body composition parameters included total body water (TBW), extracellular water (ECW), phase angle (PA) and the ECW/TBW ratio. For BIA measurement, participants lay supine with their arms positioned away from the torso and their legs slightly apart to prevent contact. Prior to the assessment, they were instructed to empty their bladder if necessary and remove any jewellery or electronic devices. The skin was cleansed and dried using an alcohol-based disinfectant without skin-conditioning additives before attaching the electrodes. Electrodes were placed dorsally on the right wrist and parallel below the knuckles of the right hand, as well as frontally on the right ankle and behind the toes, maintaining a 5-cm distance.

### Intervention

Each participant received a personalised consultation from an experienced clinical dietitian on adequate fluid intake and beverage selection. Fluid intake was determined based on Slovenian dietary guidelines, which were adapted from German Nutrition Society.^12^ To enhance understanding, the recommended fluid volume, adjusted for age and sex, was converted from millilitres into glasses, with one glass representing 200 mL. As a visual aid, each participant was provided with a colouring sheet displaying glasses, where the recommended number of glasses was pre-coloured by the clinical dietitian (online supplemental appendix A). Beverage selection was recommended based on the European Society for Paediatric Gastroenterology, Hepatology and Nutrition recommendations.^15^ Participants were advised to prioritise water, unsweetened tea and mineral water while avoiding SSBs, artificially sweetened drinks, carbonated beverages and energy drinks.^15^ Participants were required to adhere to the fluid intake recommendations for four months, after which a follow-up assessment was conducted. During questionnaire completion and delivery of the intervention, parents or guardians were present to minimise recall bias and to provide general support for adherence to the recommended fluid intake at home.

### Follow-up assessment

After four months, a follow-up assessment was conducted. If a participant had not previously undergone body composition assessment, the follow-up was completed via telephone. During this call, children and their parents answered questions regarding headaches and fluid intake. For those who had undergone body composition assessment, an in-person follow-up visit was scheduled, where questionnaires were completed, and anthropometric measurements and body composition assessments were repeated.

### Data analysis

The collected data were analysed using R (version 4.3.1), employing the tidyverse,^16^ arsenal,^17^ ggstatsplot^18^ and robustbase packages.^19^ Descriptive statistics, including arithmetic mean, median, data range and SD, were calculated. We used the Wilcoxon signed-rank test for comparative analyses and robust linear regression to assess associations between headache characteristics (dependent variable) and independent variables, such as fluid intake and type of fluid. Statistical significance was set at p<0.05.

### Statistical power calculation

Our power analysis for the Wilcoxon signed-rank test, based on a sample size of 60 and an effect size of 0.65 (Cohen’s d), showed high statistical power. Monte Carlo simulations, accounting for the skewed distribution in our data, estimated a power >99%, confirming that our study was well-powered to detect the effect of interest and minimising the risk of Type II error (false negatives).

## Results

### Participants

Initially, we aimed to recruit 76 children; however, 14 were excluded for not meeting the inclusion criteria, and two eligible patients declined participation due to the lack of time to complete the questionnaires (figure 1). No participants were lost to follow-up. At enrolment, 37 participants attended their first paediatric neurology appointment, while 23 were included during a follow-up appointment. As a result, 60 children aged 5–19 years (mean 12.7±3.4 years) were included, with 26 (43.3%) boys and 34 (56.7%) girls. The age distribution was two participants in early childhood (≥3 years to <6 years), 20 in late childhood (≥6 years to <12 years), 35 in adolescence (≥12 years to <18 years) and 3 participants in adulthood (≥18 years). The mean body weight z-score was 0.65±1.22, height z-score 0.79±1.18 and mean BMI z-score was 0.44±1.3. Of these, 29 (48.3%) had migraines, 15 (25.0%) had tension-type headaches and 16 (26.7%) had an unspecified headache type. Mean age of headache onset was 9.8±3.2 years. Mean duration of headache symptoms prior to enrolment was 2.8±2.6 years. At baseline, headaches occurred on average of 1–2 times/week, lasting 3 hours, with an intensity of 7/10 on VAS.

### Effects of the intervention

Each participant received individualised dietary counselling from a clinical dietitian on adequate fluid intake,^12^ as well as appropriate beverage selection.^15^ At the baseline, 52 participants (86.7%) met the recommended daily fluid intake, increasing to 56 participants (93.3%) post-intervention. Fluid consumption increased significantly from 5–8 glasses/day (one glass=200 mL) to 8–10 glasses/day (p<0.001).

**Figure 2:**
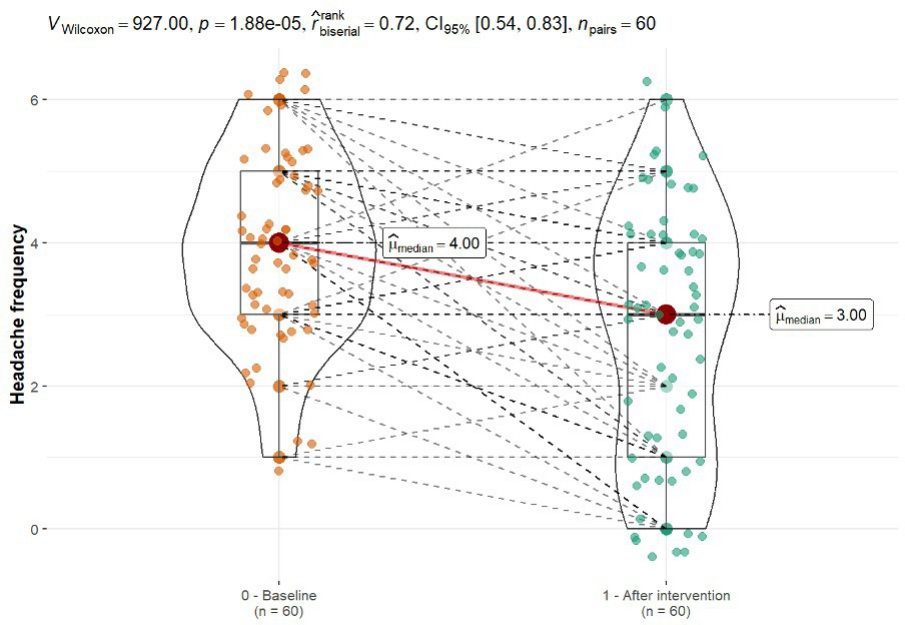
Comparison of primary headache frequency of children and adolescents at the baseline and after a four-month intervention. The results are presented using a combined chart, integrating a violin plot and a box plot. The y-axis represents primary headache frequency (0=1 time/month; 1=2 times/month; 2=3 times/month; 3=1-2 times/week; 4=3-5 times/week; 5=daily headaches). The x-axis consists of two columns: the left column (orange dots) represents each participant's primary headache frequency before the intervention, while the right column (green dots) represents the frequency after the intervention. Lines connecting the two columns depict individual changes in primary headache frequency. A downward trend indicates a reduction in primary headache frequency, whereas an upward trend signifies an increase. The red dot in the centre of each column represents the median (µ) value for the respective group.

Headache frequency significantly decreased from 1–2 times/week to 3 times/month (p<0.001), as shown in figure 2.

**Figure 3:**
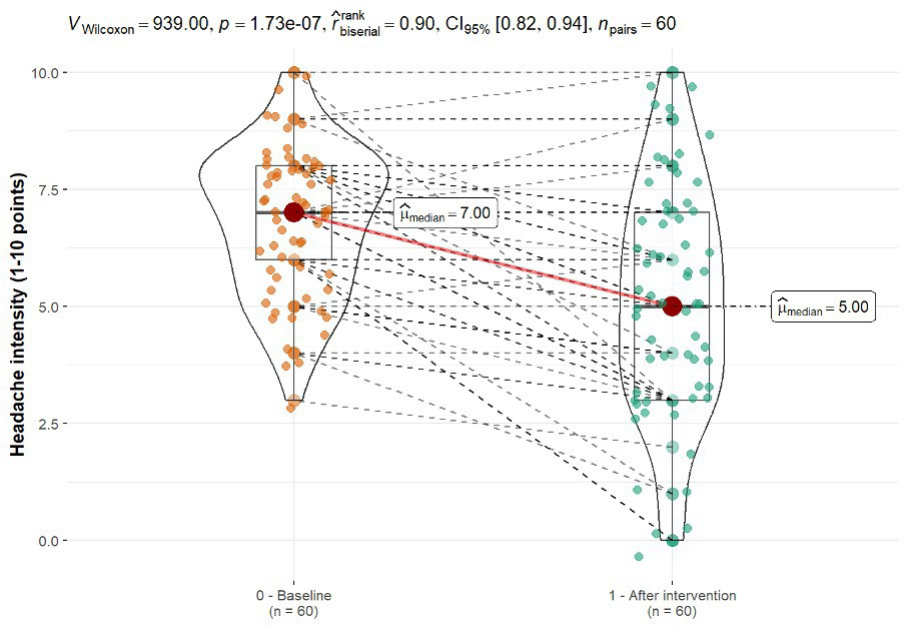
Comparison of primary headache intensity at the baseline and after a four-month intervention. The results are presented using a combined chart, integrating a violin plot and a box plot. The y-axis represents primary headache intensity, measured using a 10-point Visual Analog Scale (VAS), where 10 corresponds to the highest pain intensity. The x-axis consists of two columns: the left column (orange dots) represents each participant's primary headache intensity before the intervention, while the right column (green dots) represents the intensity after the intervention. Lines connecting the two columns illustrate individual changes in primary headache intensity. A downward trend signifies a reduction in intensity, whereas an upward trend indicates an increase. The red dot in the centre of each column represents the median (µ) value for the respective group.

Headache intensity measured using VAS declined from 7/10 at baseline to 5/10 post-intervention (p<0.001), as shown in figure 3.

**Figure 4:**
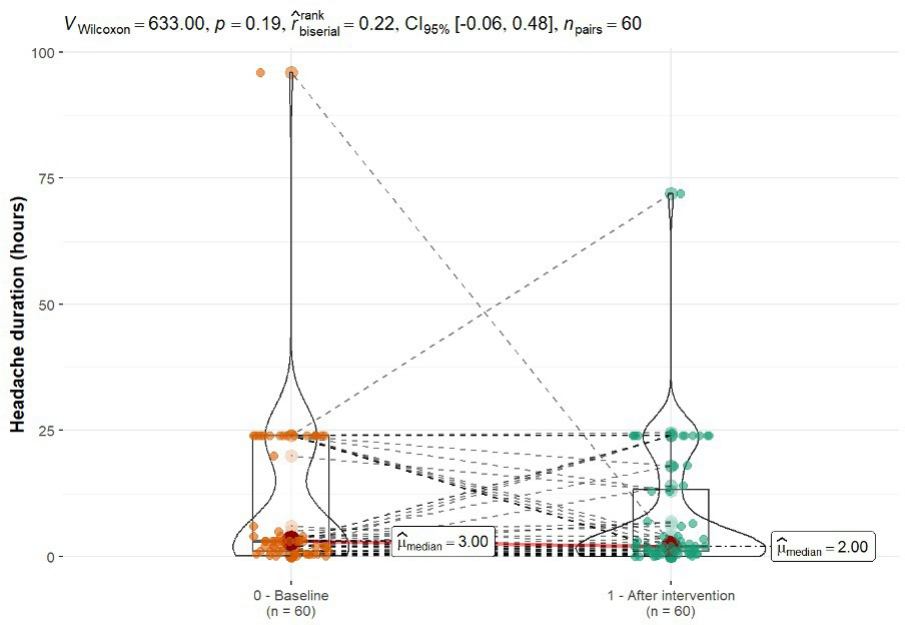
Comparison of primary headache duration at baseline and after a four-month intervention. The results are presented using a combined chart, integrating a violin plot and a box plot. The y-axis represents primary headache duration in hours. The x-axis consists of two columns: the left column (orange dots) represents each participant's headache duration before the intervention, while the right column (green dots) represents the duration after the intervention. Lines connecting the two columns illustrate individual changes in primary headache duration. A downward trend signifies a reduction in duration, whereas an upward trend indicates an increase. The red dot in the centre of each column represents the median (μ) value for the respective group.

The duration of individual headache episodes decreased from 3 hours/headache to 2 hours/headache; however, this change did not reach statistical significance (p=0.19), as shown in figure 4.

### Effect of increased water intake on hydration status

Body composition was assessed using BIA before and after the four-month intervention. To evaluate the effects of increased fluid intake on hydration status, only participants with complete pre-intervention and post-intervention BIA measurements were included (n=28; 46.7%). Following the intervention, median TBW (L) increased significantly from 29.3 L to 30.6 L (p<0.001), indicating improved hydration. Similarly, median ECW (L) increased significantly from 13.4 L to 14.3 L (p=0.007), further supporting improved hydration, while the ECW/TBW ratio (%) remained unchanged (46.5%; p=0.87). There was a non-significant change in PA (°), with median PA value increasing slightly from 5.0° to 5.2° (p=0.18). Results are shown in table 1.

**Table 1:** Changes in hydration parameters before and after a four-month intervention. Statistical significance was determined using the Wilcoxon signed-rank test (n=28).

Parameter	Baseline Median (interquartile range)	After a four-month intervention Median (interquartile range)	P value
Total body water (TBW, L)	29.3 (23.05, 33.20)	30.6 (24.30, 34.55)	<0.001
Extracellular water (ECW, L)	13.4 (9.78, 16.40)	14.3 (9.75, 16.30)	0.007
ECW/TBW ratio (%)	46.5 (44.00, 49.25)	46.5 (44.75, 48.50)	0.87
Phase angle (PA) (°)	5.0 (4.58, 5.30)	5.2 (4.60, 5.50)	0.18

### Effect of beverage choice on headaches

We aimed to examine the association between the consumption of unsweetened beverages, as recommended by dietary guidelines for hydration in children and adolescents,^15^ and the frequency, duration and intensity of headaches.

At baseline, the average frequency of unsweetened beverages consumption, as determined by the FFQ, was 1.65±0.48 (equal to 1–2 times/day or more), while the average frequency of SSBs consumption was 0.70±0.51 (equivalent to 4–6 times/week). After the intervention, the FFQ was not repeated; instead, participants self-reported which fluids they had increased during the intervention. Most participants primarily increased their intake of water (93.3%; n=56), followed by 100% fruit juices (15%; n=9), non-carbonated beverages (10%; n=6), unsweetened tea (5%; n=3), carbonated drinks (1.7%; n=1) and energy drinks (1.7%; n=1). These findings suggest a high adherence to the beverage selection recommendations.

Using robust regression, which reduces the influence of outliers, results suggest that consuming unsweetened beverages is mildly associated with fewer headaches (estimate=−0.56, p=0.085). However, robust regression showed even weaker and statistically non-significant associations with headache duration (estimate=28.84, p=0.110) and headache intensity (estimate=−0.38, p=0.191). Thus, despite employing robust methods that minimise the impact of unusual data points, the evidence remains too weak to clearly confirm a beneficial effect of unsweetened beverages on headaches.

## Discussion

This is the first interventional study to examine the impact of adequate hydration on primary headaches in a paediatric population, limiting direct comparison with similar studies in children and adolescents. In contrast, available research has focused on adults, despite dehydration being a well-recognised trigger for headaches,^2 9 11^ and adequate fluid intake being recommended as a non-pharmacological strategy for headache management in children.^5^ A pilot trial by Spigt *et al*^10^ examined the effects of increased fluid intake on migraines in 18 adults. Participants in the intervention group were instructed to increase their daily fluid intake by 1500 mL for 12 weeks. By the end of the study, headache duration in the intervention group decreased by 21 hours over 2 weeks compared with the control group (95% CI: −48 to −5), though this reduction was not statistically significant. Similarly, in our study, the average headache duration decreased from 3 to 2 hours, but this change was also not statistically significant. Spigt *et al*^10^ reported a reduction in headache intensity, measured using a 100-mm VAS (−13 mm; 95% CI: −32 to −5). In our study, headache intensity, assessed with a 10-point VAS, significantly decreased from 7/10 at baseline to 5/10 post-intervention (p<0.001). Additionally, while Spigt *et al*^10^ found no significant effect of fluid intake on headache frequency, our study demonstrated a significant reduction in headache frequency from 1–2 times/week to three times/month (p<0.001). In a larger, randomised, placebo-controlled study involving 102 adults with primary headaches, Spigt *et al*^7^ again investigated the effect of increasing daily fluid by 1500 mL over 3 months. However, they did not find a significant reduction in headache frequency or duration, which partially contradicts our findings. One possible explanation is that participants in the Spigt *et al*^7^ study increased their fluid intake by only 842 mL on average, which may not have been sufficient to exert a significant effect on headache parameters. In contrast, our participants increased their fluid intake from 5–8 glasses/day (1000–1600 mL) to 8–10 glasses/day (1600–2000 mL), resulting in a statistically significant difference (p<0.001).

### Hydration and headache prevention in paediatric populations

There is currently no published research specifically investigating the effect of increased fluid intake on headaches in children and adolescents. However, studies have confirmed insufficient fluid intake in the paediatric population. In a study by Kroon Van Diest *et al*,^20^ adolescents with migraines consumed an average of 5±3 glasses of fluid/day, which is below the recommended 8–10 glasses/day. Similarly, in our study, pre-intervention fluid intake was 5–8 glasses/day, which increased to 8–10 glasses/day post-intervention (p<0.001). The findings of both studies emphasise the importance of addressing inadequate hydration in children and adolescents.

Although 86.7% of participants in our cohort reported the recommended daily fluid intake at baseline, this finding should be interpreted cautiously. Self-reported adequacy does not necessarily reflect true hydration status: in the BIA subgroup (n=28), TBW and ECW increased significantly after the intervention, suggesting a real physiological improvement despite reported baseline adequacy. Moreover, reported intake may not capture beverage quality. Some participants consumed SSBs at baseline, and the intervention specifically encouraged water and unsweetened drinks, which may have contributed to the observed clinical improvements. Finally, reliance on the FFQ may lead to recall bias and overestimation of baseline intake.

Taken together, these considerations highlight the complexity of assessing hydration status and support the importance of promoting adequate, high-quality fluid intake as a simple, non-pharmacological strategy for headache prevention in clinical practice.

### Hydration and body composition in paediatric populations

The effectiveness of our intervention was further supported by improvements in hydration status, measured by BIA. Both the median TBW and ECW increased (p<0.001 and p<0.007, respectively), indicating enhanced overall hydration. Furthermore, the ECW/TBW ratio remained unchanged and statistically insignificant, suggesting that fluid distribution remained stable, maintaining fluid balance without signs of fluid retention or shifts between compartments. This stability in ECW/TBW further reinforces the physiological relevance of the observed hydration improvements. Additionally, the median PA showed a slight increase (p=0.18), suggesting a potential improvement in cellular membrane integrity and hydration. Overall, these findings demonstrate a positive effect of adequate fluid intake on body hydration, reinforcing the effectiveness of our intervention.

### Hydration and beverage choice in paediatric population

Kenney *et al*^21^ assessed hydration status in 4134 American children and adolescents (52.7% boys, 47.3% girls; aged 6–19 years) using urinary osmolality. They found that 54.5% were inadequately hydrated, with boys at a significantly higher risk than girls (p<0.001). The mean daily water intake was 2.9 servings, which was significantly associated with lower urinary osmolality and a reduced risk of dehydration. In contrast, the mean daily intake of SSBs was 2.0 servings, correlating with higher urinary osmolality. The study concluded that water intake is more effective in improving hydration than other beverages, while SSBs may promote dehydration due to their high sugar content, which increases urine output.^22^ These findings align with our results, where consuming unsweetened beverages was associated with a non-significant reduction in headache duration and frequency. Additionally, moderate water consumption has no known adverse effects, whereas SSB intake has been linked to an increased risk of metabolic syndrome, obesity, type 2 diabetes and cardiovascular diseases.^15 21^

In a cross-sectional study by Guelinckx *et al*^23^ involving 3611 children (4–9 years) and 8109 adolescents (10–17 years), the total fluid intake was 1200 mL/day in both groups. Similar findings were reported in the Slovenian National Food Consumption Survey ‘SI.Menu 2017/18’,^24^ which included 468 adolescents (49% boys, 51% girls) aged 10–17 years, where the average total fluid intake was reported to be 1600 mL/day. The results of both studies are comparable to the baseline fluid intake in our study, where children and adolescents aged 5–19 years consumed 1000–1600 mL/day. Following the intervention, fluid consumption increased to 1600–2000 mL/day. However, it is important to highlight that the proportion (%) of participants in SI.Menu 2017/18 who consumed below recommended daily intake of water and non-alcoholic beverages was 57% for girls and 61% for boys aged 10–13 years, and 61% for girls and 60% for boys aged 14–17 years.^24^ A similar trend was observed in a study by Martinez *et al*,^25^ which found that 61% of children and 75% of adolescents failed to meet the adequate intake recommendations for water from fluids. These findings further emphasise the importance of improving hydration habits among children and adolescents, with a focus on increasing and promoting the consumption of water and unsweetened beverages. Considering the potential effects of inadequate hydration on health, particularly its link to headaches, it is essential to implement targeted efforts to support better hydration practices in children and adolescents.

### Strengths and limitations of the study

Our study is the first to examine the impact of hydration and adequate fluid intake on headaches in children and adolescents. The broad age range of participants (5–19 years) provides valuable insight into the effects of hydration across different stages of childhood and adolescence, enhancing the generalisability of our findings across the paediatric population.

Our sample size provided high statistical power and enhanced the validity of our conclusions regarding differences between pre-intervention and post-intervention measurements.

However, certain limitations should be acknowledged. The study was not randomised and did not include a control group, which limits the ability to determine whether the observed changes were solely due to the intervention or influenced by other confounding factors, such as analgesic use. The study was conducted between May and September, during warmer months when hydration might play a more prominent role than in cooler months—conducting the study year-round would help overcome this limitation. Additionally, while hydration status was objectively assessed using BIA, this was only conducted on a subset of 28 participants, which may have limited statistical power.

Data collection was based on self-reported questionnaires, where participants retrospectively assessed the frequency, intensity and duration of headaches, potentially introducing recall bias. A more precise approach would involve the use of a headache diary over a specified period to enhance data accuracy. Similarly, fluid intake was estimated using a FFQ rather than a fluid intake diary, which may have led to measurement errors, as intake was reported in glasses. However, parental verification of reported intake helped mitigate potential bias. Finally, while participants were given advice about the maximum frequency of use of analgesics, we did not record their use.

As part of routine clinical care, participants received general lifestyle advice from the paediatric neurologist (eg, maintaining regular meals, obtaining adequate sleep, engaging in physical activity and reducing screen time). Although these recommendations were broad and not accompanied by specific quantitative targets, they may still introduce potential bias, as changes in lifestyle habits could partially influence headache outcomes. Only hydration counselling was structured, personalised and quantitatively defined, which helps isolate the primary focus of the intervention. One of our limitations is only monitoring compliance with the recommended fluid intake through self-report. To minimise recall bias, parents were present during reporting to help verify daily fluid consumption. In addition, the subgroup of participants who underwent pre-intervention and post-intervention BIA measurements provided partial objective validation of adherence to the intervention through observed changes in hydration-related parameters.

### Implications for future research or clinical practice

Our findings enhance the understanding of the impact of fluid intake and hydration on the duration, frequency and intensity of headaches in children and adolescents, contributing to the development of more effective clinical management strategies. Future interventional studies could further improve compliance monitoring by using digital tools, such as mobile applications or daily tracking calendars in which participants record the number of glasses consumed each day, as well as incorporating additional biomarkers of hydration status (eg, urine osmolality, plasma osmolality). The doubly labelled water method could also be used as an objective biomarker of total water turnover and hydration status.

Additionally, it would be valuable to explore the impact of hydration on different subtypes of primary headaches (eg, migraines, tension-type headaches) and to investigate the impact of hydration on analgesic use.

Lifestyle factors such as sleep, meal regularity, physical activity and screen time were collected through the baseline questionnaire; however, the present manuscript focuses specifically on hydration and its association with headache outcomes. Future studies should further investigate the independent and combined effects of lifestyle factors on headache burden in children and adolescents.

## Conclusion

Our study is the first to examine hydration as a potential factor in preventing and managing primary headaches in paediatric patients. Our findings suggest that increasing daily fluid intake and improving beverage choices may be associated with headache improvement. Clinicians should encourage proper hydration habits and discourage the consumption of SSBs to improve both headache outcomes and overall health. Regular monitoring of hydration status and body composition may offer valuable insights into patient care, further enhancing treatment effectiveness. Given the potential role of hydration on headache frequency and intensity, integrating hydration-focused strategies into clinical practice represents a simple, cost-effective and non-pharmacological intervention for paediatric headache management. Future research should explore the long-term effects of hydration interventions and potential mechanisms linking fluid intake with headache pathophysiology. These findings reinforce the need for multidisciplinary approaches that incorporate dietary and lifestyle modifications alongside conventional treatment strategies to optimise patient outcomes.

## Supplementary material

10.1136/bmjnph-2025-001341 online supplemental appendix 1

## Data Availability

Data are available upon reasonable request.
