# Supplementary material for: Hydration and primary headaches in children and adolescents: a prospective interventional study
Source: BMJ Nutr Prev Health. 2026 Jan 19;9(1):e001341. doi: 10.1136/bmjnph-2025-001341 (PMC13425120; doi:10.1136/bmjnph-2025-001341)
Supplement: online supplemental appendix 1 [file bmjnph-9-1-s001.pdf]

NAME:________________________________

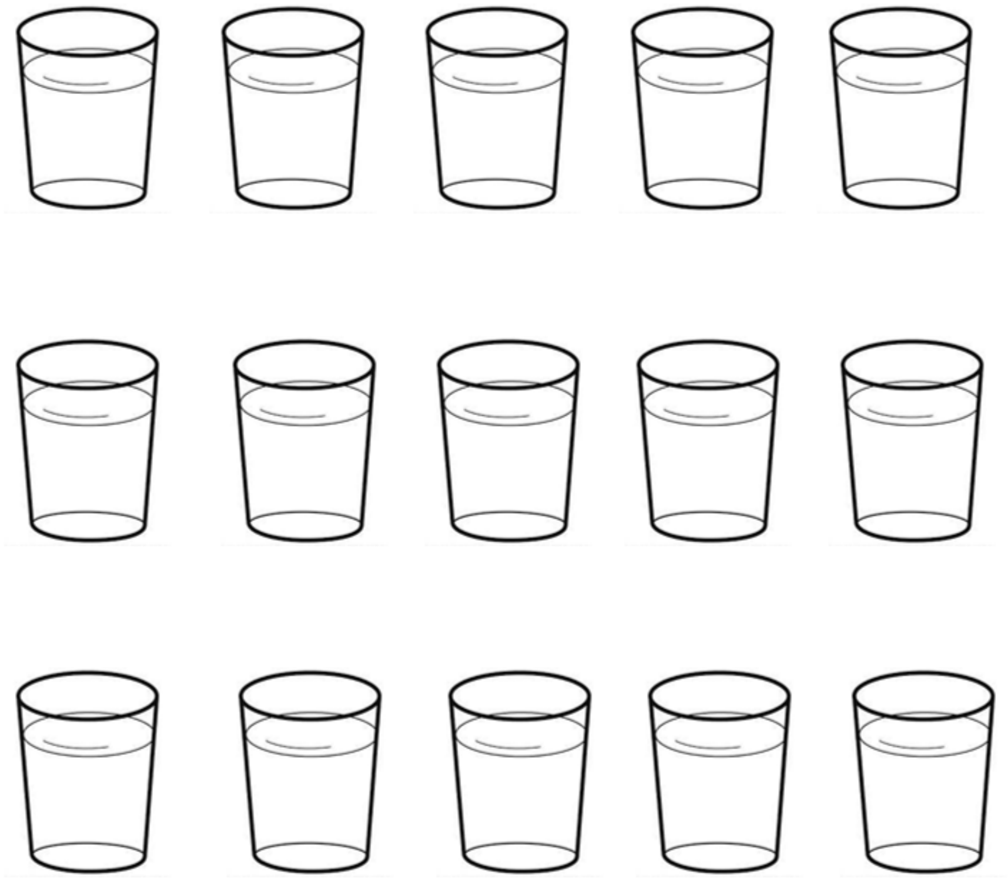

Appendix A Colouring chart used to provide individualized recommendations for daily fluid intake (1 glass=200 mL).
